# Risk factors for death from pandemic influenza in 1918–1919: a case–control study

**DOI:** 10.1111/irv.12228

**Published:** 2014-02-03

**Authors:** Jennifer A Summers, James Stanley, Michael G Baker, Nick Wilson

**Affiliations:** a Division of Health and Social Care Research, King's College LondonLondon, UK; b Department of Public Health, University of Otago WellingtonWellington, New Zealand

**Keywords:** 1918–1919 Influenza pandemic, infectious disease, influenza, military, pandemic

## Abstract

**Background:**

Despite the persisting threat from future influenza pandemics, much is still unknown about the risk factors for death from such events, and especially for the 1918–1919 influenza pandemic.

**Methods:**

A case–control study was performed to explore possible risk factors for death from pandemic influenza among New Zealand military personnel in the Northern Hemisphere in 1918–1919 (*n* = 218 cases, *n* = 221 controls). Data were compiled from a Roll-of-Honour dataset, a dataset of nearly all military personnel involved in the war and archived individual records.

**Results:**

In the fully adjusted multivariable model, the following were significantly associated with increased risk of death from pandemic influenza: age (25–29 years), pre-pandemic hospitalisations for a chronic condition (e.g. tuberculosis), relatively early year of military deployment, a relatively short time from enlistment to foreign service, and having a larger chest size (e.g. adjusted odds ratio for 90–99 cm versus <90 cm was 2·45; 95% CI=1·47–4·10). There were no significant associations in the fully adjusted model with military rank, occupational class at enlistment, and rurality at enlistment.

**Conclusions:**

This is one of the first published case–control studies of mortality risk factors for the 1918–1919 influenza pandemic. Some of the findings are consistent with previous research on risk factors (such as chronic conditions and age groups), but others appear more novel (e.g., larger chest size). As all such historical analyses have limitations, there is a need for additional studies in other settings as archival World War One records become digitalised.

## Introduction

The 1918–1919 H1N1 influenza pandemic, which caused the death of an estimated 50–100 million people worldwide, represents one of the worst pandemics, of any kind, experienced by humans in recorded history. This pandemic occurred during the final stages of World War One (WW1), and the transportation of large numbers of troops worldwide probably assisted its spread over the course of several months.^1^

The 1918–1919 pandemic had a range of epidemiological similarities to the most recent H1N1 pandemic starting in 2009. For example, higher mortality risk in both pandemics has been associated with lower socio-economic status, being a young adult, being pregnant, being a member of an indigenous or ethnic minority, and/or having a pre-existing condition such as a chronic respiratory illness.^1^–^10^

Nevertheless, much remains unknown about risk factors for mortality from pandemic influenza. Such information could facilitate pandemic planning and optimal use of control measures during future pandemics, such as vaccination. Consequently, our study aimed to examine possible risk factors for the 1918–1919 pandemic in a well-defined population. This was the military personnel in the New Zealand Expeditionary Force (NZEF) of WW1. As a group, this force represented around 40% of the New Zealand adult male population during 1918–1919. There has been previous research on other military populations exposed to the 1918–1919 pandemic which has identified various pre-enlistment variables and combat-related exposures as risk factors for increased mortality.^1^,^4^,^5^,^9^,^11^,^12^ Yet very little previous work has had access to individualised data sources that is now becoming available as military archives are digitalised and made publicly available.

## Methods

We aimed to conduct a case–control study with cases and controls being military personnel located in the Northern Hemisphere (mainly Europe). The focus on just this hemisphere was because of the differing exposures to pandemic waves worldwide (including number of waves), and varying aspects related to military service (such as varying discharge procedures after Armistice, and conditions).^1^,^4^,^5^,^7^,^9^,^11^–^14^ For example, the military personnel in New Zealand had much higher mortality rates than those in the Northern Hemisphere, possibly owing to most of them being new recruits and residing in crowded military camps. The pandemic period for the Northern Hemisphere was defined (based on historical records and documentation detailed in an unpublished PhD thesis^11^,^13^) as occurring from 27 August 1918 until 31 March 1919.

Potential cases were identified through the use of an electronic dataset (Roll-of-Honour) covering all deaths amongst NZEF personnel in WW1, as per a previous study.^12^ This electronic dataset was obtained by courtesy of the compiler, Professor Peter Dennis (Australian Defence Force Academy). Cases were defined as those whose specified cause of death was one of the following: influenza, pneumonia and/or bronchitis during the defined Northern Hemisphere pandemic period (as per the above dates). Details in this dataset cover military records, background information and details regarding place and cause of death.

Controls were randomly selected from an electronic database of New Zealand military personnel participating in WW1 obtained from the Cenotaph database compiled by the Auckland War Memorial Museum,^15^ as per a previous study.^12^ This dataset is freely available online to the public: http://muse.aucklandmuseum.com/databases/cenotaph/locations.aspx (although we purchased a copy from the Auckland Museum in Excel format). Initial control sample selection included 1000 records (approximately 1% of the 100 000 records in the Cenotaph database). The Cenotaph dataset contains details of war service, and basic demographic data. All potential cases, as identified in the Roll-of-Honour database, were cross-referenced in the Cenotaph database for consistency and accuracy.

Selection criteria required all cases and controls (as initially identified in the Roll-of-Honour or Cenotaph databases) to have a digitised PDF military record available with the required information for analysis. This information included demographic, anthropometrics, medical and military details. These military records were either accessed onsite at a New Zealand military camp (permission obtained in January 2011 from the NZ Defence Force) or were accessed online via the Archives New Zealand website (http://www.archway.archives.govt.nz). As all this information is in the public domain (and all NZEF personnel are deceased), there were no privacy restrictions relating to these data and no requirement for additional ethical review. The need for the PDF of the military file was due to the additional variables/information required as part of this study and because much of this information was missing from both the Roll-of-Honour and Cenotaph datasets.

Specific details on how data were classified follow:

### Socio-demographic and anthropometric factors

Data were collected on age, height and weight. From these data, body mass index (BMI) was calculated using height and weight measurements. Measurements of chest size (recorded as part of medical and uniform requirements upon enlistment^16^) were also collected. It is assumed that these ‘minimum’ and ‘maximum’ measurements reflect chest circumference at the peaks of forced exhalation and inspiration; however, archival military records are not clear on this matter. Ethnicity classification was based on a coding system detailed elsewhere (and which include the language for names/parents’ names and home address, for example, if from the Pacific Islands).^10^ Occupation at enlistment was used to classify occupation status along with an online occupational coding system developed from a large New Zealand historical study^17^ (http://www.caversham.otago.ac.nz/electors/erform.php). Different degrees of rurality (pre-enlistment) were identified as detailed in a previous study by the authors^12^). The measures of rurality consist of the following: city, large town (or large county and town), small town (or small county and town) and rural.

### Military factors

Final WW1 military rank was classified as in previous studies,^12^,^18^ along with the date of enlistment, deployment/embarkment from New Zealand to Europe via troopship and length of military service (derived variable).

### Pandemic/medical factors

These data included total number of hospitalisations during military service (from 1914 until the end of the relevant pandemic period: 31 March 1919), and aspects of hospitalisations occurring before the Northern Hemisphere pandemic period (recorded admission/diagnosis for respiratory [e.g. tuberculosis], chronic [e.g. venereal disease] or wounds).

### Statistical analysis

Data checking was performed in MS Excel 2007, and statistical analysis was performed using the following: Epi-Info (Centers for Disease Control and Prevention, Atlanta, GA, USA), Open-Epi (http://www.OpenEpi.com, Accessed 6 April 2013) and sas version 9.1 (SAS Institute Inc., Cary, NC, USA). A two-sided *P*-value of <0·05 was used to indicate statistical significance.

Multivariable logistic modelling was used (Proc Logistic in SAS), as the outcome was binary: that is, either died or did not die from pandemic influenza. The strategy for the modelling was sequential, so each set of factors was included in each subsequent model, of which there were four in the final adjusted multivariable model.

Each model (and the specific exposure factors) was based on both the outcome of the univariate results and previous literature. The first model focused on host variables such as age and aspects of body size. Model two explored specific pandemic and medical factors (combined with model one). The third model introduced military aspects, which is particularly important given that the sample was a military population (combined with models one and two). The fourth model introduced socio-demographic variables to give the final ‘fully adjusted’ model.

For each categorical variable included in the multivariable analysis, the type three analysis of effect *P*-value (or factor *P*-value) was included to assess group rather than single parameters. During the process of adding variables to the multivariable models, problems with co-linearity were checked (none were found).

## Results

### Participants and descriptive data

A total of 218 cases (63% of all cases who died in the Northern Hemisphere) and 221 controls (22% of the initial *n* = 1000 control sample) fitted the inclusion criteria (Table 1). The average ages at the start of the pandemic period for cases and controls were 28·3 and 28·1 years of age, respectively.

**Table 1:** Characteristics of pandemic influenza deaths amongst cases

Variable	Number (*n* = 218)	Per cent (%)
Stated cause of death		
Influenza	99	45·0
Pneumonia	118	54·0
Bronchitis	1	0·0
Time in hospital before death (days)		
0–9	141	65·0
10–19	48	22·0
20–29	10	5·0
30–39	4	2·0
40+	12	6·0
Unknown	3	1·0
Mean days in hospital before death	11·4	–
Median days in hospital before death	7	–
Region of death		
United Kingdom/Ireland	86	39·0
Belgium/France	75	34·0
Middle East (Egypt, Iran, Palestine and Turkey)	35	16·0
Other European country (Germany and Switzerland)	22	10·0

### Univariate results

Enlistment variables associated with a statistically increased mortality risk in the univariate analysis were as follows (Table 2): being aged 25–29 years (compared with under 25 year olds); chest maximum circumference of 90–99 cm or 100+ cm (compared with under 90 cm), listing a large town as enlistment address (compared with a city enlistment address); a probable rural occupation (when compared to other occupations); and a final rurality score between one and six (when compared to an ‘all urban’ score of zero).

**Table 2:** Univariate analysis of risk factors for mortality from the 1918 to 1919 influenza pandemic amongst the WW1 NZEF

Variables*	Cases (*n* = 218)	Controls (*n* = 221)	Unadjusted odds ratio (95% CI)
	number (per cent)		
Age at start of pandemic period (years)			
Under 25	68 (31·0)	89 (40·0)	1·0 reference
25–29	70 (32·0)	52 (24·0)	**1·76 (1·09–2·84)**
30–34	46 (21·0)	40 (18·0)	1·51 (0·89–2·55)
35+	34 (16·0)	40 (18·0)	1·11 (0·64–1·94)
Chi-square for linear trend = 0·40 (*P* = 0·527)			
Mean age (years)	28·31	28·07	–
Median age (years)	28	26	Kruskal–Wallis (KW) test = 1·23 (*P* = 0·267)
Ethnicity			
European/other	211 (97·0)	217 (98·0)	1·0 reference
Māori/Pacific peoples	7 (3·0)	4 (2·0)	1·8 (0·52–6·24)
Height (m)			
	70 (32·0)	90 (41·0)	1·0 reference
1·7–1·79	120 (55·0)	109 (49·0)	1·41 (0·94–2·12)
1·8+	28 (13·0)	22 (10·0)	1·64 (0·86–3·10)
Chi-square for linear trend = 3·22 (*P* = 0·07)			
Mean height (m)	1·72	1·71	–
Median height (m)	1·71	1·71	KW test = 1·92 (*P* = 0·166)
Weight (kg)			
45–54	8 (4·0)	12 (5·0)	1·0 reference
55–64	77 (35·0)	100 (45·0)	1·16 (0·45–2·97)
65–74	106 (49·0)	75 (34·0)	2·12 (0·83–5·44)
75+	27 (12·0)	34 (15·0)	1·19 (0·43–3·33)
Chi-square for linear trend = 1·83 (*P* = 0·18)			
Mean weight (kg)	67·22	66·38	–
Median weight (kg)	67·13	64·41	KW test = 2·60 (*P* = 0·107)
Body mass index (BMI)			
Underweight (<18·5 BMI)	3 (1·0)	8 (4·0)	0·36 (0·09–1·38)
Normal (18·5–24·9 BMI)	189 (87·0)	181 (82·0)	1·0 reference
Overweight (25–29·9 BMI)	24 (11·0)	28 (13·0)	0·82 (0·46–1·47)
Obese (BMI of 30 or greater)	2 (1·0)	4 (2·0)	0·48 (0·09–2·65)
Chi-square for linear trend = 0·16 (*P* = 0·69)			
Mean BMI	22·69	22·65	–
Median BMI	22·56	22·43	KW test = 0·256 (*P* = 0·613)
Minimum chest circumference (cm)			
Under 80	27 (12·0)	34 (15·0)	1·0 reference
80–89	153 (70·0)	149 (67·0)	1·29 (0·74–2·25)
90+	38 (17·0)	38 (17·0)	1·26 (0·64–2·48)
Chi-square for linear trend = 0·27 (*P* = 0·60)			
Mean minimum chest circumference (cm)	85·44	84·91	–
Median minimum chest circumference (cm)	85·09	83·82	KW test = 1·725 (*P* = 0·189)
Maximum chest circumference (cm)			
Under 90	43 (20·0)	77 (35·0)	1·0 reference
90–99	154 (71·0)	126 (57·0)	**2·19 (1·41–3·40)**
100+	21 (10·0)	18 (8·0)	**2·09 (1·01–4·34)**
**Chi-square for linear trend = 8·70 (*****P*** **= 0·003)**			
Mean maximum chest circumference (cm)	93·82	92·76	–
Median maximum chest circumference (cm)	93·98	91·44	**KW test = 5·473 (*****P*** **= 0·019)**
Chest circumference difference (cm)			
Under 5	18 (8·0)	36 (16·0)	1·0 reference
5–9	165 (76·0)	164 (74·0)	1·38 (0·44–4·32)
10+	35 (16·0)	21 (10·0)	2·43 (0·75–7·93)
**Chi-square for linear trend = 8·73 (*****P*** **= 0·003)**			
Mean chest circumference difference (cm)	8·38	7·85	–
Median chest circumference difference (cm)	7·62	7·62	KW test = 3·647 (*P* = 0·056)
Occupational class (pre-enlistment)			
1–3 (highest occupational class)	8 (4·0)	14 (6·0)	1·0 reference
4–6	86 (39·0)	87 (39·0)	1·73 (0·69–4·33)
7–9 (lowest occupational class)	124 (57·0)	120 (54·0)	1·81 (0·73–4·47)
Chi-square for linear trend = 0·72 (*P* = 0·40)			
Mean occupational class	6·54	6·53	–
Median occupational class	7	7	KW test = 0·004 (*P* = 0·948)
Rural occupation measure			
Rural occupation (e.g. farmer)	59 (27·0)	58 (26·0)	1·12 (0·73–1·72)
Probably rural occupation (e.g. fencer)	20 (9·0)	10 (5·0)	**2·20 (1·00–4·87)**
Other occupations	139 (64·0)	153 (69·0)	1·0 reference
Chi-square for linear trend = 0·64 (*P* = 0·42)			
Rural location			
City	80 (37·0)	100 (45·0)	1·0 reference
Large town (or large county and town)	51 (23·0)	22 (10·0)	**2·90 (1·62–5·18)**
Small town (or small county and town)	44 (20·0)	66 (30·0)	0·83 (0·52–1·35)
Rural	43 (20·0)	33 (15·0)	1·63 (0·95–2·80)
Chi-square for linear trend = 0·53 (*P* = 0·50)			
Final rurality score (combination of location and occupation as per the two above items)			
0 score (urban)	56 (26·0)	84 (38·0)	1·0 reference
1–2	70 (32·0)	57 (26·0)	**1·84 (1·13–3·00)**
3–4	41 (19·0)	34 (15·0)	**1·81 (1·03–3·19)**
5–6	37 (17·0)	29 (13·0)	**1·91 (1·06–3·46)**
7–8 (highly rural)	14 (6·0)	17 (8·0)	1·24 (0·57–2·71)
Chi-square for linear trend = 2·53 (*P* = 0·11)			
Mean final rurality score	2·69	2·43	–
Median final rurality score	2	2	KW test = 2·43 (*P* = 0·119)
Final military rank achieved			
Officers/Non-Commissioned Officers	50 (23·0)	48 (22·0)	1·07 (0·68–1·68)
Others	168 (77·0)	173 (78·0)	1·0 reference
First deployment/embarkment year			
1914–1916	113 (53·0)	57 (26·0)	**3·24(1·86–5·64)**
1917	75 (34·0)	115 (52·0)	1·06 (0·62–1·83)
1918	30 (14·0)	49 (22·0)	1·0 reference
**Chi-square for linear trend = 25·45,** ***P*** **= <0·001**			
Time from enlistment until foreign service (deployment/embarkment)			
Under 4 months	125 (57·3)	72 (32·6)	1·0 reference
4–8 months	75 (34·0)	114 (52·0)	**0·38 (0·25–0·57)**
8 months+	18 (8·0)	35 (16·0)	**0·30 (0·16–0·56)**
Chi-square for linear trend = 31·80 (*P* < 0·001)			
Mean time (days)	142·45	175·1	–
Median time (days)	115·5	147	**KW test = 25·872 (*****P*** **< 0·001)**
Time from enlistment up to pandemic period			
Under 2 years	99 (45·0)	146 (66·0)	1·0 reference
2–3 years	71 (33·0)	66 (29·0)	**1·59 (1·04–2·42)**
3 years+	48 (22·0)	10 (5·0)	**7·08 (3·42–14·65)**
**Chi-square for linear trend = 30·24 (*****P*** **< 0·001)**			
Mean time (days)	820	648·76	–
Median time (days)	791	602	**KW test = 22·726 (*****P*** **< 0·001)**
Ever wounded			
No	203 (93·0)	218 (99·0)	1·0 reference
Yes	15 (7·0)	3 (1·0)	**5·37 (1·53–18·82)**
Total number of hospitalisations during entire military service (to either death or end of pandemic period)			
0	15 (7·0)	51 (23·0)	1·0 reference
1–2	138 (63·0)	138 (62·0)	**3·40 (1·83–6·33)**
3+	62 (28·0)	32 (14·0)	**6·59 (3·22–13·49)**
Unclear	3 (1·0)	0 (0·0)	–
**Chi-square for linear trend = 26·97 (*****P*** **< 0·001)**			
Mean number of hospitalisations	1·95	1·35	–
Median number of hospitalisations	2	1	**KW test = 27·207 (*****P*** **< 0·001)**
Total number of hospitalisations during military service during the pre-pandemic period			
0	89 (41·0)	70 (32·0)	1·0 reference
1–2	102 (47·0)	129 (58·0)	**0·62 (0·41–0·93)**
3+	24 (11·0)	22 (10·0)	0·86 (0·44–1·66)
Unclear	3 (1·0)	0 (0·0)	–
Chi-square for linear trend = 2·17 (*P* = 0·141)			
Mean number of hospitalisations	1·05	1·15	–
Median number of hospitalisations	1	1	KW test = 2·472 (*P* = 0·116)
Pre-pandemic respiratory admission**			
No	198 (91·0)	192 (87·0)	1·0 reference
Yes	20 (9·0)	29 (13·0)	0·67 (0·37–1·22)
Pre-pandemic chronic condition admission***			
No	181 (83·0)	200 (90·0)	1·0 reference
Yes	37 (17·0)	21 (10·0)	**1·95 (1·10–3·45)**

* Bolded values signify statistical significance (*P* < 0·05).

** Respiratory hospitalisations were defined as the following: influenza, pneumonia, bronchitis, pyrexia of unknown origin, trench fever and cough (not further defined).

*** Chronic condition hospitalisations were defined as illnesses/conditions, which are persistent and require (in particular for this time period) long-term management. They included the following (as identified in this sample): previously gassed, venereal disease, malaria, cardiac conditions, pleurisy, colitis, tuberculosis, nephritis, asthma, trench feet, shingles, oedema and periostitis.

Comparison of military and pandemic/medical variables found the following factors to be significantly associated with increased mortality risk: first deployment year between 1914 and 1916 (compared with 1918), <4 months from enlistment until foreign service, time from enlistment to pandemic period of ‘2–3 years and 3 years+’ (compared with under 2 years), any prior hospitalisation for wounds during military service, pre-pandemic hospitalisation for a chronic condition and total number of hospitalisations during entire military service at one or more (compared with zero hospitalisations).

### Multivariable modelling results

Being aged 25–29 years at the start of the pandemic period was independently associated with increased mortality risk in all four models (Table 3). In all four models, a larger maximum chest circumference relative to the reference group of ‘under 90 cm’ was independently associated with an increased mortality risk. This is in contrast to the other anthropometric measure in the final analysis, BMI, which showed no association with mortality risk.

**Table 3:** Multivariable analysis of risk factors for mortality from the 1918 to 1919 influenza pandemic amongst the WW1 NZEF

Northern Hemisphere models***		Adjusted odds ratio (95% CI)							

		Model 1 (age/anthropometric)	Factor *P*-value	Model 2 (model 1 + medical)	Factor *P*-value	Model 3 (model 2 + military)	Factor *P*-value	Model 4 (model 3 + socio-demographic)	Factor *P*-value
Reference	Variables								
Age at start of pandemic period (years)									
<25 years	25–29	**1·85 (1·13–3·03)**	0·143	**1·96 (1·18–3·25)**	0·094	**1·68 (1·01–2·92)**	0·343	**1·78 (1·01–3·15)**	0·275
	30–34	1·41 (0·81–2·43)		1·42 (0·81–2·49)		1·38 (0·75–2·53)		1·37 (0·75–2·52)	
	35–39	1·11 (0·56–2·19)		1·14 (0·57–2·29)		1·21 (0·55–2·52)		1·17 (0·55–2·46)	
	40+	0·97 (0·43–2·19)		0·89 (0·39–2·06)		0·80 (0·33–1·97)		0·82 (0·33–2·02)	
Maximum chest circumference (cm)									
Under 90	90–99	**2·26 (1·43–3·57)**	**0·002**	**2·41 (1·50–3·87)**	**0·001**	**2·57 (1·55–4·27)**	**0·001**	**2·45 (1·47–4·10)**	**0·002**
	100+	**2·39 (1·05–5·45)**		**2·59 (1·12–6·00)**		**2·97 (1·21–7·29)**		**2·89 (1·16–7·20**)	
Body mass index (BMI)									
Normal	Underweight	0·40 (0·10–1·58)	0·122	0·32 (0·08–1·27)	0·071	0·28 (0·06–1·28)	**0·041**	0·27 (0·06–1·26)	**0·045**
	Overweight/obese	0·60 (0·32–1·11)		0·58 (0·31–1·09)		0·51 (0·25–1·01)		0·51 (0·25–1·01)	
Total number of hospitalisations during military service before pandemic period									
0	1–2		**0·47 (0·30–0·74)**	**0·004**	**0·32 (0·19–0·53)**	**<0·001**	**0·32 (0·19–0·54)**	**<0·001**
	3+		0·71 (0·32–1·56)		0·47 (0·20–1·12)		0·43 (0·18–1·03)	
Pre-pandemic chronic condition admission									
No	Yes			**2·22 (1·17–4·18)**		**2·00 (1·03–3·90)**		**2·02 (1·02–3·97)**	
Previous respiratory admission before pandemic period									
No	Yes			0·78 (0·39–1·57)		0·74 (0·35–1·59)		0·74 (0·35–1·59)	
First deployment/embarkment year									
1918 (last year of WW1)	1914–1916					**3·26 (1·57–6·79)**	**<0·001**	**3·40 (1·62–7·16)**	**<0·001**
	1917					1·16 (0·62–2·18)		1·17 (0·62–2·21)	
Final military rank									
Others	Officers/NCOs					0·71 (0·42–1·20)		0·73 (0·42–1·27)	
Time from enlistment until foreign service (deployment/embarkment)									
<4 months	4–8 months					**0·53 (0·32–0·88)**	**0·03**	**0·55 (0·33–0·92)**	0·054
	8+ months					0·48 (0·23–1·02)		0·51 (0·23–1·10)	
Occupational class (pre-enlistment)									
1–3	4–6							0·84 (0·27–2·66)	0·857
	7–9							0·77 (0·25–2·38)	
Final rurality score									
0 (urban)	1–2							1·69 (0·97–2·95)	0·242
	3–4							1.72 (0.90–3.29)	
	5–6							1.87 (0.95–3.67)	
	7–8 (highly rural)							1·29 (0·52–3·17)	

* Each of the multivariable models is added sequentially to the final adjusted model (4).

** Bolded values signify statistical significance (*P* < 0·05).

Statistically significant associations were found in relation to pandemic and medical-related variables. Those with one to two total pre-pandemic hospitalisations during military service had a significantly decreased mortality risk. Whilst the factor *P*-value was significant in all models, having had three or hospitalisations was not significantly associated with mortality risk. Any hospitalisation for a pre-pandemic chronic condition was independently associated with an increased mortality risk in all models. However, hospitalisations for pre-pandemic respiratory conditions were not associated with mortality risk.

A first deployment/embarkment year occurring between 1914 and 1916 was independently associated with an increased mortality risk, whilst this was not so for those embarking in 1917. The factor *P*-value for this variable was significant, including after adjusting for socio-demographic factors. Final military rank was not significantly associated with mortality risk in any model.

Longer time between deployment/enlistment until foreign service was associated with a decreased mortality risk. However, this association was only significant for those serving 4–8 months prior to the pandemic. The final ‘fully adjusted’ model (model four) factor *P*-value was not significant. The measures of occupational class and rurality were not found to be independently associated with mortality risk in this final model.

## Discussion

### Main findings and interpretation

**Figure 1:**
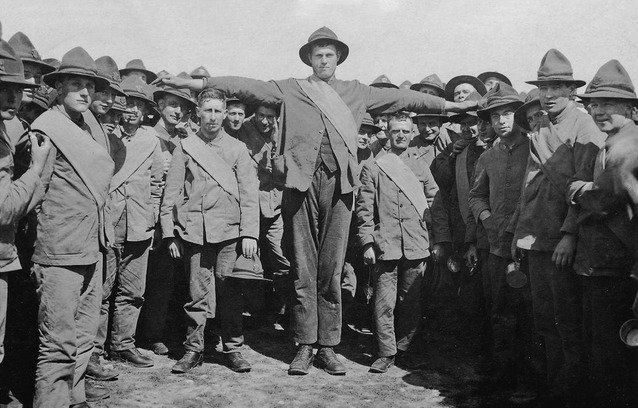
A group of unidentified WW1 NZ soldiers. Taken in 1917, in an NZ military training camp prior to deployment/embarkment to Europe. From private collection. Of note is that this case–control study found that large chest size, as per the man in the middle, was independently associated with risk of pandemic influenza mortality.

One of the notable findings of this study is around larger chest size and increased risk of pandemic-related mortality. More specifically, the maximum chest circumference, potentially an indicator of maximum vital lung capacity, showed a consistent significant association with increased mortality risk, in both the univariate and multivariable analyses (see Figure 1 for examples of the range of body builds among these military personnel). This could still be a chance finding, but if true, then it may suggest a differential immune system response to pandemic influenza infection in larger men (e.g. possibly an increased chance of a cytokine storm response). This finding fits in with anecdotal observations related to larger individual size and increased risk of complication and/or death during 1918^1^,^19^ and evidence from the subsequent 2009 influenza pandemic.^2^,^20^–^22^ Furthermore, there was some evidence for influence of other measures of body size (apart from chest measurements) on pandemic mortality risk. The unadjusted odds ratios (ORs) for both height and weight, whilst not statistically significant, do suggest some possible degree of association between increased height and weight with mortality risk. It is also plausible that the large chest size may have reflected chronic asthma (a fairly established risk factor for more recent studies of influenza-related complications^3^,^21^) in some of the personnel. Nevertheless, data on the prevalence of asthma during this period and in this military cohort are not well described; therefore, it was not able to be accessed in this study. Similarly, although smoking was common in this cohort (based on photographs of the soldiers), we suspect that most would not have been smoking long enough to have developed measurably impaired respiratory capacity by the time of the pandemic.

An increased mortality risk for those with a first deployment/embarkment year prior to 1917 was found in both the univariate and multivariable analyses. This finding is in contrast to other research that suggests that ‘fresh troops’ were at an increased risk of pandemic mortality.^11^,^23^–^25^ However, this association may be the result of the selection process of this study excluding those who travelled during the pandemic period or were located in the Southern Hemisphere. It may also be the result of an unknown cohort effect, with earlier personnel being volunteers, and later personnel a mixture of volunteers and conscripts.

The association between length of service time and mortality risk may reflect the impact of chronic psychological and physical stress, which may have impaired immune responses (along with possible roles from nutrient deficiencies which were identified in some NZEF personnel^26^, and co-existing illnesses). This hypothesis is further supported by the increased mortality risk associated with pre-pandemic chronic disease hospitalisations in the final adjusted model. It is worth noting that an increased time from enlistment until foreign service (deployment/embarkment) was associated with a decreased mortality risk. This could reflect increased pathogen exposure in the military environment providing acquired immunity and suitable recovery time before deployment to the combat environment.

The findings for an association between pre-pandemic hospitalisations and mortality risk in this study are complex to interpret. Having a pre-pandemic hospitalisation for a chronic condition was consistently a risk factor for increased pandemic mortality risk in both the univariate and multivariable analyses. However, having one to two pre-pandemic hospitalisations (any cause) was associated with a decreased risk of mortality. One possible explanation is that personnel with prior hospitalisations may have been exposed to pathogens whilst in hospital that may have provided some form of acquired immunity (at least to bacterial agents causing secondary pneumonia) during the subsequent pandemic. But this effect may have been outweighed by the vulnerability associated with more serious chronic conditions (i.e. reflecting a more weakened immune system), as suggested by other researchers.^25^ However, this argument is speculative, and future research would be required to explore these issues further.

Being wounded at any time during service was found to be associated with a subsequent risk of death from pandemic influenza. This is notable given that the ‘wounded’ classification in the military records included exposure to chemical warfare agents (e.g. to mustard gas) and lung damage due to gas exposure is known to result in chronic detrimental health effects.^27^,^28^

Pandemic mortality risk varied by age group amongst personnel in Europe in both the univariate and multivariable analyses. An increased mortality risk was significant amongst personnel aged 25–29 years when compared to those under 25 years, similar to previous studies.^1^,^5^,^12^,^14^,^25^,^29^,^30^ The bimodal shaped curve of pandemic mortality in relation to age is still evident in both the unadjusted and adjusted ORs.

A finding in the univariate analysis was an association between mortality risk and the measure of rurality, although this was not statistically significant in the multivariable model. If some level of association exists, these rurality results may reflect the interaction between past exposure to pathogens and potential acquired immunity in a subsequent influenza outbreak. For example, during the 2009 influenza pandemic, those born before 1954 were found to have some form of immunity, resulting in lower morbidity and mortality rates than expected.^3^,^31^,^32^ Interestingly, it is personnel for whom their pre-enlistment rurality is judged to be a semi-rural background (large town or large county and town) that experienced an increased mortality risk, somewhat consistent with previous research.^9^ This complicated finding may be due to increased past pathogen exposure (with greater population exposure), and resulting acquired immunity amongst urban personnel, whilst the rural personnel may have been healthier through potentially higher-quality nutrition prior to military service. However, this is all relatively speculative given the limitations of this historical data.

### Strengths and limitations

This study is probably only the second published case–control study to consider mortality risk factors from the 1918 to 1919 influenza pandemic (following the one by Shanks *et al*.^25^). It is also amongst the most detailed analytic studies of this pandemic as it includes individual-level anthropometric data, detailed rurality coding and time-period-specific occupational class coding.

Whilst caution must be maintained in trying to generalise the results from both an extremely virulent pandemic and a military population to modern day pandemic scenarios, the NZEF personnel of WW1 represented around 40% of the New Zealand male population of military age in 1918 (around 10% of the total New Zealand population). Therefore, the military records from the NZEF personnel of WW1 can potentially provide meaningful information with regard to outcomes during the 1918–1919 influenza pandemic.

An important limitation of this study is that selection of cases and controls was restricted to those present in the Northern Hemisphere during the entire pandemic period. As this period coincides with the cessation of hostilities (11 November 1918), it introduces a possible bias in terms of personnel who were sent back to New Zealand during the pandemic period. For example, the earliest NZEF cohorts to leave Europe were a combination of those who had served the longest and those who were unwell (i.e. wounded or suffering from illness). However, this may also mean that both the cases and controls included in this study were potentially healthier than those travelling or those discharged back to New Zealand prior/during the Northern Hemisphere pandemic period. Consequently, the selection for this sample is probably biased towards a survivor effect. The survivor effect could work in many ways in this sample. For example, it has been suggested that soldiers were less likely to be shot if they were shorter than average (i.e. being ‘killed in action’ may have been correlated with height).^33^ Alternatively, the sample could potentially be less healthy compared with earlier cohorts (many of whom were not present in the Northern Hemisphere, and not included in this study). That is, it is possible that later NZEF cohorts were less healthy due to down-grading of medical restrictions in the later stages of WW1 for New Zealanders.^1^

The case definition of a pandemic-related death in this study was fairly broad; however, this was necessary given the lack of laboratory confirmation available, as there are no known surviving pathological specimens from this military cohort. Consequently, it is not possible to separate the effect of secondary bacterial infections from influenza infection on its own. However, the definition adopted in this study is consistent with other studies from the period, which regard all reported influenza, pneumonia and bronchitis deaths among exposed personnel during the pandemic period as pandemic-related mortality.^1^,^14^,^16^,^25^ The peaked nature of the pandemic's epidemic curve for these military forces^13^ also strongly suggests that virtually all disease-related deaths during the defined ‘pandemic period’ were influenza related.

The exclusion of potential cases and controls based on incomplete military files also presents an important methodological problem in this study. For example, military files of those located in Belgium/France (therefore in a combat-active location) may be less complete than those located in the UK. Unfortunately, there is no reliable way to ascertain whether this is a strong selection bias for this study.

Initially, it was decided that if information differed significantly between the Roll-of-Honour and Cenotaph databases and the online PDFs of military files, reselection of cases and controls would be undertaken. However, this did not occur as all key variables for which data were collected (e.g. age, occupation, military rank) were identical. This finding supports the high accuracy/quality between the different sources of information. Future research could probably rely on just one source of information, rather than a combination of databases and PDFs of military files.

## Conclusions

The results of this study suggest that a combination of host and environmental factors (e.g. as indicated by military variables) played a role in determining pandemic mortality risk amongst the NZEF personnel located in Europe during the 1918–1919 pandemic period. The role of variations in age, chest size and occurrence of chronic disease, in determining mortality risk, has implications for modern-day risk assessment, particularly as these variables are not modifiable when a pandemic emerges (though chronic disease can be prevented to some degree). For example, in a pandemic scenario, limited health resources will need to be prioritised to certain populations groups estimated to be at greater risk of morbidity and mortality. A useful valid way of identifying these higher-risk populations is through the study of past pandemics, although understanding the impact of seasonal influenza may also help. More research on these risk factors for the 1918–1919 pandemic will become increasingly possible as records from WW1 become digitalised.

This study has suggested the negative effect of a long military service prior to the 1918–1919 influenza pandemic. If supported by other studies, this result may also have implications for future military populations in the wake of an infectious disease outbreak. Furthermore, this finding emphasises the impact of health status during a pandemic (e.g. chronic disease as a risk factor for serious illness and/or mortality during an influenza pandemic).

The 1918–1919 influenza pandemic represents one of the worst pandemics, of any kind, experienced by humans in recorded history. It could therefore be described as a worst case scenario for guiding future population-based pandemic planning. Therefore, understanding the great lethality of this pandemic is relevant to preparations for future pandemics, given the recurrent nature of influenza pandemics, the substantial burden on the human population and the associated potential burden on limited healthcare resources. Indeed, relative to the 2009 pandemic, future pandemics could be much more serious, highlighting the on-going need to assess past pandemics for future pandemic planning and health sector resourcing.
